# Tuberculin skin test conversion in patients under treatment with anti-tumor necrotizing factor alpha agents

**DOI:** 10.1186/s12879-020-05166-5

**Published:** 2020-07-02

**Authors:** Mohammad-Esmaeil Hejazi, Atefeh Ahmadzadeh, Alireza Khabbazi, Aliasghar Ebrahimi, Maryam Farmani, Yasin Hejazi

**Affiliations:** 1 Internal medicine department, Tabriz University of Medical Sciences, Tabriz, Iran; 2 Connective Tissue Diseases Research Center, Tabriz University of Medical Sciences, Tabriz, Iran

**Keywords:** Tuberculin skin test, Anti-TNF-alpha agents, Tuberculosis

## Abstract

**Background:**

Despite successful clinical outcomes of biologic medications in patients with chronic rheumatic diseases, some considerable adverse effects such as infections remain a major concern. Possibility of tuberculosis (TB) reactivation over treatment with anti-tumor necrotizing factor (TNF) alpha agents has necessitated a screening test before initiation of treatment. However, screening over the course of treatment is not recommended in those patients with negative baseline screening tests. This study aimed to evaluate the efficacy of tuberculin skin test (TST) before treatment in patients with chronic rheumatologic diseases who were indicated to receive anti-TNF-alpha therapy and the necessity of repeating this test over the course of treatment.

**Methods:**

In this prospective study, patients with chronic rheumatologic diseases receiving anti-TNF-alpha agents were studied in a two-year period. TST was performed before treatment and those with positive results were excluded from the study. Thereafter, treatment with anti-TNF-alpha agents was initiated with the indicated dose. TST was repeated before administration of biologic treatment until TST became positive or 16 weeks after the initiation of treatment with anti-TNF-alpha.

**Results:**

A total of 51 cases were studied, of whom one patient (1.9%) was excluded due to positive TST before treatment. All participants received infliximab and the TST test became positive in one patient (2%) 2 weeks after receiving the first dose. Also, the results of further tests at weeks 6, 10, and 14 were all negative for the remaining patients.

**Conclusion:**

Due to the possibility of TST conversion after administration of anti-TNF-alpha therapy, it is important to consider TB monitoring in patients under treatment with these agents using available methods such as TST.

## Background

The Community Oriented Program for Control of Rheumatic Diseases (COPCORD) and the International League of Associations for Rheumatology (ILAR) by the collaboration of the World Health Organization (WHO) revealed that rheumatic complains were the commonest complaint in the community, and soft tissue rheumatism, ill-defined musculoskeletal symptoms, and osteoarthritis were the most prevalent disorders [1]. The urban COPCORD study in developing countries such as Iran demonstrated that in the population over the age of 15 years rheumatic complains were seen in 41.9% of people. Degenerative joint disease and inflammatory disorders were also reported in a considerable proportion of patients [2]. Different therapeutic options have been recommended for rheumatologic diseases, such as non-steroidal anti-inflammatory drugs, traditional disease-modifying anti-rheumatic drugs (DMARDs), and glucocorticoids [3, 4]. Moreover, numerous biologic therapies have emerged in the recent decades with significantly successful outcomes, including tumor necrosis factor-alpha (TNF-alpha) blockers, CTLA4-Ig, anti-interleukin I (IL-1) and anti-IL 6 receptors, and rituximab (an anti CD20 antibody) [5–7]. However, some complications, particularly infections, are not uncommon by using these medications, both as a direct consequence of the treatment or due to the underlying disease process [8–10]. Reactivation of tuberculosis (TB) has also been widely reported in patients receiving biologic therapies, in particular anti-TNF-alpha agents [11–13]. Therefore, tuberculin skin test (TST) or interferon-gamma release assay (IGRA) is strictly recommended before the initiation of therapy [13]. Most current guidelines and expert reviews recommend that in case of the absence of risk factors and clinical suspicion for TB, there is no need for repeating TB screening tests [13, 14]. However, there are some reports of TB infection in patients under treatment with biologic therapies and negative TST at initiation [15–17]. These reports raise the concern about the inadequacy of a single TST test before initiation of treatment. However, no prospective study has been conducted in this regard. Therefore, we aimed to evaluate the efficacy of TST before treatment in patients with chronic rheumatologic diseases who were indicated to receive anti-TNF-alpha therapy and necessity of repeating this test over the course of treatment.

## Methods

**Fig. 1:**
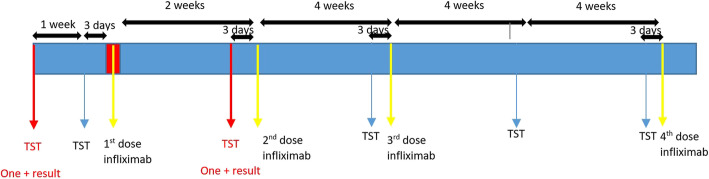
Timing of the administration of infliximab and tuberculin skin test (TST)

This prospective observational study was conducted on patients (in any age or sex) with a chronic rheumatologic disease referred to Imam Reza Teaching Hospital of Tabriz University of Medical Sciences for receiving anti-TNF-alpha agents in a two-year period (March 2017 to March 2019). Patients were excluded if they had a medically confirmed history of active or latent TB infection, household TB contact, or unevaluated symptoms that could possibly be due to TB infection, such as chronic cough. Informed consent was obtained from all participants. TST was performed 10 days before treatment with the standard method by an internal medicine specialist and was confirmed by another internal medicine specialist. Patients with positive TST tests were excluded from the study and referred to the TB control centers for further diagnostic and/or therapeutic procedures. The study was continued with TST negative patients. One week later, TST test was repeated and the positive tests were considered as booster effect; these cases were also excluded from the study. Thereafter, treatment with anti-TNF-alpha agents was initiated with the indicated dose. Infliximab was administered at weeks 0, 2, and 6 and then every 8 weeks. The timing of the TST test and infliximab administrations are illustrated in Fig. 1. TST was repeated by the same person with the same procedure before administration of treatment until TST became positive or 16 weeks after the initiation of treatment with anti-TNF-alpha.

TST was done by an internal specialist on the volar side of the left forearm with the Mantoux method. Ten units of tuberculin purified protein derivative (PPD) was injected intradermally and the injected site was marked (PPD RT23; Staten Serum Institute, Copenhagen, Denmark). The appearance of any induration was evaluated 72 h after injection using the ballpoint method [18]. The same procedure was repeated each time the TST was performed. An induration of more than 5 mm was considered in patients receiving immunosuppressive drugs, such as methotrexate or cyclosporine; otherwise, 10 mm or higher was considered positive. Also, any increase in diameter of induration of TST was defined as positive TST [19, 20].

### Statistical analysis

Continuous data were reported as mean and standard deviation. The frequency and percentage of qualitative variables were also reported. SPSS version 24 was used for all analyses.

## Results

A total of 51 patients participated in this study, out of whom one patient with ankylosing spondylitis (AS) and a positive TST before initiation of treatment was excluded from the study. The study was conducted on 50 patients, including 28 males (56%) and 22 females (44%). The mean age was 31.2 ± 6.55 years (range: 20–50 years). Also, 33 patients (66%) had AS and 17 patients (34%) had rheumatoid arthritis (RA). Concurrent use of methylprednisolone was reported in 17 (34%) patients (Table 1). All patients had received Bacillus Calmette–Guérin (BCG) vaccination in their childhood.

**Table 1:** Characteristics of included patients

Total number	50
Age (mean ± SD years)	31.2 ± 6.6
≤ 18 (n, %)	0
18–39 (n, %)	42 (84%)
40–49 (n, %)	7 (14%)
≥ 50 (n, %)	1 (2%)
BCG vaccination at birth (n, %)	50 (100%)
Type of disease	
Ankylosing spondylitis	33 (66%)
Rheumatoid arthritis	17 (34%)
Biologic treatment	
Infliximab (n, %)	50 (100%)
Each dose (mg)	200
Other treatments	
Indomethacin (n, %)	13 (26%)
Daily dose (mg)	150
Naproxen (n, %)	4 (8%)
Daily dose (mg)	1500
Diclofenac (n, %)	16 (32%)
Daily dose (mg)	200
Hydroxychloroquine (n, %)	17 (34%)
Daily dose mean ± SD dose (mg)	270.5 ± 98.5
Methotrexate (n, %)	17 (34%)
Weekly dose mean ± SD dose (mg)	13.8 ± 3.3
Prednisolone	
Daily dose mean ± SD (mg)	5.29 ± 1.21
≤ 2.5 mg (n, %)	1 (2%)
= 5 mg (n, %)	13 (26%)
≥ 7.5 mg (n, %)	3 (6%)
No prednisolone (n, %)	33 (66%)

All patients with negative TST included in our study received infliximab with the standard dose. Before administration of the second dose of infliximab (2 weeks after the first dose of infliximab), a male 37-year-old patient with AS developed a positive TST (induration, 8 mm; Table 2). The TST induration of this patient prior to biologic treatment was 3 mm; he received indomethacin concomitantly but did not receive prednisolone or other non-steroidal anti-inflammatory drugs. Moreover, he had no household TB contact. The patient was referred to TB control center for further evaluation and the study continued with the remaining patients. However, no other positive TST cases were seen when TST was repeated in the following weeks. In addition, none of the patients had symptoms of TB.

**Table 2:** TST results

#	Time of test	Number of tested patients	Result	
1#	10 days before treatment	51	Positive *	1 (1.9%)
			Negative	50 (98.1%)
2#	3 days before treatment	50	Positive	0
			Negative	50 (100)
3#	3 days before the second dose	50	Positive *	1 (2%)
			Negative	49 (98%)
4#	3 days before the third dose	49	Positive	0
			Negative	49 (100)
5#	Four weeks after the third dose	49	Positive	0
			Negative	49 (100)
6#	3 days before fifth dose	49	Positive	0
			Negative	49 (100)

TST positive patients were referred to TB control centers and TB infection was confirmed

## Discussion

The possibility of TB reactivation by anti-TNF-alpha treatment has been well-established by several studies, and guidelines recommend performing screening tests before initiation of these drugs. However, the majority of current guidelines suggest that there is no need for re-screening TB infection after initiation of biologic treatments [14]. We evaluated the sufficiency of TST in patients with chronic rheumatic diseases indicated to receive biologic therapy. Our results demonstrated that there is a possibility of positive TB infection after administration of biologic drugs despite a negative prior screening test (conversion of TST) that can be detected by repeating TST over the course of treatment. Although this was seen only in one over 50 patients included in the study, neglecting this finding and poor detection and management of this highly communicable disease can lead to terrible consequences.

Several studies have revealed the risk of TB infection in patients who receive TNF-alpha inhibitors [21–23]. Askling et al. investigated the Swedish Inpatient Register RA cohort (62.321 patients) and reported that 230 individuals in this cohort were diagnosed with TB during the 14 years follow-up period, of whom, 15 patients had received TNF-alpha inhibitors (11 patients were treated with infliximab) [24]. A conversion from negative TST to positive after treatment with anti-TNF-alpha has also been reported by some studies. Park et al. reported a considerable ratio of 32.6% of patients having a conversion from negative to positive TST by using biologic medications [17]. Also, Slouma et al. reported that there were two cases of active pulmonary TB among patients receiving anti-TNF-alpha therapy with initially negative TST and QuantiFERON-TB Gold test [16]. Another study by Mobini et al. also reported a case of seropositive RA under treatment with infliximab that got an active TB infection despite a previous negative TB screening test [15]. This finding has also been reported for patients with non-rheumatic diseases. Celine Debeuckelaere et al. reported two patients with chronic inflammatory bowel disease (IBD) that developed a TB infection after treatment with anti-TNF-alpha agents, despite a negative screening test [25]. Also, a Korean study reported de novo TB infection in 3.1% of IBD patients after anti-TNF-alpha therapy [26].

A panel of experts recommend that annual TB screening test should be considered in patients with RA, AS, psoriatic arthritis (PsA), or psoriasis under treatment with anti-TNF-alpha agents if they travel or work in situations where TB exposure is likely regardless of negative screening test at baseline [13]. However, this has not been adequately appreciated in the current guidelines.

There are two predominant screening tests for TB including TST and IGRA. Despite well-known false-negative and false-positive TST results, the standard screening test is still TST along with a comprehensive medical history and chest X-ray [27]. Furthermore, TST is simpler, has lower costs, and is a widely available test. Therefore, in our study we did not perform IGRA and only TST was conducted as a screening test. Meanwhile, we attempted to diminish the disadvantages of TST; for example, TST was administrated meticulously by an expert and under supervision to reduce the negative impact of misperformance. We did not have any false-positive results as both TST-positive patients (before and after treatment) were confirmed to have active TB by further evaluations. Nonetheless, we could not roll out false-negative TST in our patients due to their immunosuppression treatment. Oral prednisolone is reported to have some impact on TST results; however, this impact is predominantly dose dependent [28]. Kleinert et al. and Ponce de Leon et al. demonstrated that 7.5–10 mg/day may impair TST results [29]. However, majority of our patients received prednisolone with a dose of equal or less than 5 mg/day. Regarding the patient who presented positive TST after treatment, it is unlikely that the negative TST before treatment was due to immunosuppression because he was not under treatment with immunosuppressive drugs and he received the medications with the same dose along with infliximab.

A conversion in TST, defined as a change from negative to a positive test, can occur when a new or enhanced hypersensitivity arise due to de novo TB infection or non-TB mycobacteria, including BCG vaccination [19]. This reaction has been variedly reported to occur 3 to 7 weeks after exposure [19]. In our study, positive TST was seen after 2 weeks of baseline TST (2 weeks after initiation of treatment). This could be due to the booster effect; however, considering that we conducted a second TST 3 days before treatment to roll out this phenomenon, a booster effect was also unlikely to be considered for our patient.

TNF-alpha has an important role in both the host immune response to TB infection and in its immunopathology [30]. It is produced by a variety of immune cells in response to various pathogens, such as lipopolysaccharide or viral and bacterial infections [31]. TNF-alpha in response to TB infection brings about several positive effects. The main receptor of TNF-alpha, acting against TB infection is TNF receptor 1 (TNFR1) [32]. In vitro studies have demonstrated that TNFR1 is essential in both granuloma formation and in susceptibility to intracellular pathogens during TB infection. This results in controlling the mycobacteria and preventing their dissemination [30]. Therefore, it is conceivable that inhibition of this mediator by anti-TNF-alpha agents leads to poor immune reaction potency against TB infection.

The global prevalence of RA is more than AS; but in our study 66% of patients who received infliximab were AS. The main reason was our center’s strategy for treatment of RA. We use biologics after failing combination therapy with three DMARDs for controlling the disease activity. However, some rheumatologists use rituximab as the first biologic for treating seropositive RA.

Our study had some limitations. It was better to perform IGRA and chest X-ray for more comprehensive screening of the patients. But due to our center’s protocol and some aforementioned reasons we only performed TST. Moreover, due to the relatively small number of patients in our study we found only one positive TST after initiation of treatment. It could be possible to detect more positive cases in a larger sample size.

## Conclusion

Our study demonstrated the possibility of TST conversion (positive TST) after the administration of infliximab. Therefore, it is important to consider re-screening TB in patients receiving infliximab after initiation of the treatment even if the screening tests were negative before treatment.

## Data Availability

All Data and material are available upon request.

## Abbreviations

TNF: Anti-tumor necrotizing factor
PPD: Purified protein derivative
TB: Tuberculosis
TST: Tuberculin skin test
TNFR1: TNF receptor 1
